# Cancer Risks Associated With *BRCA1* and *BRCA2* Pathogenic Variants

**DOI:** 10.1200/JCO.21.02112

**Published:** 2022-01-25

**Authors:** Shuai Li, Valentina Silvestri, Goska Leslie, Timothy R. Rebbeck, Susan L. Neuhausen, John L. Hopper, Henriette Roed Nielsen, Andrew Lee, Xin Yang, Lesley McGuffog, Michael T. Parsons, Irene L. Andrulis, Norbert Arnold, Muriel Belotti, Åke Borg, Bruno Buecher, Saundra S. Buys, Sandrine M. Caputo, Wendy K. Chung, Chrystelle Colas, Sarah V. Colonna, Jackie Cook, Mary B. Daly, Miguel de la Hoya, Antoine de Pauw, Hélène Delhomelle, Jacqueline Eason, Christoph Engel, D. Gareth Evans, Ulrike Faust, Tanja N. Fehm, Florentia Fostira, George Fountzilas, Megan Frone, Vanesa Garcia-Barberan, Pilar Garre, Marion Gauthier-Villars, Andrea Gehrig, Gord Glendon, David E. Goldgar, Lisa Golmard, Mark H. Greene, Eric Hahnen, Ute Hamann, Helen Hanson, Tiara Hassan, Julia Hentschel, Judit Horvath, Louise Izatt, Ramunas Janavicius, Yue Jiao, Esther M. John, Beth Y. Karlan, Sung-Won Kim, Irene Konstantopoulou, Ava Kwong, Anthony Laugé, Jong Won Lee, Fabienne Lesueur, Noura Mebirouk, Alfons Meindl, Emmanuelle Mouret-Fourme, Hannah Musgrave, Joanne Ngeow Yuen Yie, Dieter Niederacher, Sue K. Park, Inge Sokilde Pedersen, Juliane Ramser, Susan J. Ramus, Johanna Rantala, Muhammad U. Rashid, Florian Reichl, Julia Ritter, Andreas Rump, Marta Santamariña, Claire Saule, Gunnar Schmidt, Rita K. Schmutzler, Leigha Senter, Saba Shariff, Christian F. Singer, Melissa C. Southey, Dominique Stoppa-Lyonnet, Christian Sutter, Yen Tan, Soo Hwang Teo, Mary Beth Terry, Mads Thomassen, Marc Tischkowitz, Amanda E. Toland, Diana Torres, Ana Vega, Sebastian A. Wagner, Shan Wang-Gohrke, Barbara Wappenschmidt, Bernhard H. F. Weber, Drakoulis Yannoukakos, Amanda B. Spurdle, Douglas F. Easton, Georgia Chenevix-Trench, Laura Ottini, Antonis C. Antoniou

**Affiliations:** ^1^ Center for Epidemiology and Biostatistics, Melbourne School of Population and Global Health, The University of Melbourne, Parkville, Victoria, Australia; ^2^ Center for Cancer Genetic Epidemiology, Department of Public Health and Primary Care, University of Cambridge, Cambridge, United Kingdom; ^3^ Precision Medicine, School of Clinical Sciences at Monash Health, Monash University, Clayton, Victoria, Australia; ^4^ Department of Molecular Medicine, Sapienza University of Rome, Rome, Italy; ^5^ Harvard T.H. Chan School of Public Health, Boston, MA; ^6^ Dana-Farber Cancer Institute, Boston, MA; ^7^ Department of Population Sciences, Beckman Research Institute of City of Hope, Duarte, CA; ^8^ Department of Clinical Genetics, Odense University Hospital, Odence, Denmark; ^9^ Department of Genetics and Computational Biology, QIMR Berghofer Medical Research Institute, Brisbane, Queensland, Australia; ^10^ Fred A. Litwin Center for Cancer Genetics, Lunenfeld-Tanenbaum Research Institute of Mount Sinai Hospital, Toronto, ON, Canada; ^11^ Department of Molecular Genetics, University of Toronto, Toronto, ON, Canada; ^12^ Department of Gynaecology and Obstetrics, University Hospital of Schleswig-Holstein, Campus Kiel, Christian-Albrechts University Kiel, Kiel, Germany; ^13^ Institute of Clinical Molecular Biology, University Hospital of Schleswig-Holstein, Campus Kiel, Christian-Albrechts University Kiel, Kiel, Germany; ^14^ Service de Génétique, Institut Curie, Paris, France; ^15^ Paris Sciences Lettres Research University, Paris, France; ^16^ Division of Oncology and Pathology, Department of Clinical Sciences Lund, Lund University, Lund, Sweden; ^17^ Department of Medicine and Huntsman Cancer Institute, University of Utah Health, Salt Lake City, UT; ^18^ Departments of Pediatrics and Medicine, Columbia University, New York, NY; ^19^ Sheffield Clinical Genetics Service, Sheffield Children's Hospital, Sheffield, United Kingdom; ^20^ Department of Clinical Genetics, Fox Chase Cancer Center, Philadelphia, PA; ^21^ Molecular Oncology Laboratory, CIBERONC, Hospital Clinico San Carlos, IdISSC (Instituto de Investigación Sanitaria del Hospital Clinico San Carlos), Madrid, Spain; ^22^ Nottingham Clinical Genetics Service, Nottingham University Hospitals NHS Trust, Nottingham, United Kingdom; ^23^ Institute for Medical Informatics, Statistics and Epidemiology, University of Leipzig, Leipzig, Germany; ^24^ Division of Evolution and Genomic Sciences, School of Biological Sciences, Faculty of Biology, Medicine and Health, University of Manchester, Manchester Academic Health Science Center, Manchester, United Kingdom; ^25^ North West Genomics Laboratory Hub, Manchester Center for Genomic Medicine, St Mary's Hospital, Manchester University NHS Foundation Trust, Manchester Academic Health Science Center, Manchester, United Kingdom; ^26^ Institute of Medical Genetics and Applied Genomics, University of Tübingen, Tübingen, Germany; ^27^ Department of Gynecology and Obstetrics, University Hospital Düsseldorf, Heinrich-Heine University Düsseldorf, Düsseldorf, Germany; ^28^ Molecular Diagnostics Laboratory, INRASTES, National Center for Scientific Research “Demokritos”, Athens, Greece; ^29^ Aristotle University of Thessaloniki School of Medicine, Thessaloniki, Greece; ^30^ Department of Medical Oncology, German Oncology Center, Limassol, Cyprus; ^31^ Clinical Genetics Branch, Division of Cancer Epidemiology and Genetics, National Cancer Institute, Bethesda, MD; ^32^ Department of Human Genetics, University Würzburg, Würzburg, Germany; ^33^ Department of Dermatology, Huntsman Cancer Institute, University of Utah School of Medicine, Salt Lake City, UT; ^34^ Center for Familial Breast and Ovarian Cancer, Faculty of Medicine and University Hospital Cologne, University of Cologne, Cologne, Germany; ^35^ Center for Integrated Oncology (CIO), Faculty of Medicine and University Hospital Cologne, University of Cologne, Cologne, Germany; ^36^ Molecular Genetics of Breast Cancer, German Cancer Research Center (DKFZ), Heidelberg, Germany; ^37^ Southwest Thames Regional Genetics Service, St George's Hospital, London, United Kingdom; ^38^ Breast Cancer Research Programme, Cancer Research Malaysia, Subang Jaya, Selangor, Malaysia; ^39^ Institute of Human Genetics, University Hospital Leipzig, Leipzig, Germany; ^40^ Institute of Human Genetics, University of Münster, Münster, Germany; ^41^ Clinical Genetics Department, Guy's and St Thomas' NHS Foundation Trust, London, United Kingdom; ^42^ Faculty of Medicine, Department of Human and Medical Genetics, Institute of Biomedical Sciences, Vilnius University, Vilnius, Lithuania; ^43^ State Research Institute Center for Innovative Medicine, Vilnius, Lithuania; ^44^ Genetic Epidemiology of Cancer Team, Inserm U900, Paris, France; ^45^ Institut Curie, Paris, France; ^46^ Mines ParisTech, Fontainebleau, France; ^47^ Department of Epidemiology and Population Health, Stanford University School of Medicine, Stanford, CA; ^48^ Division of Oncology, Department of Medicine, Stanford University School of Medicine, Stanford, CA; ^49^ Department of Obstetrics and Gynecology, David Geffen School of Medicine, University of California at Los Angeles, Los Angeles, CA; ^50^ Department of Surgery, Daerim Saint Mary's Hospital, Seoul, South Korea; ^51^ Hong Kong Hereditary Breast Cancer Family Registry, Hong Kong; ^52^ Department of Surgery, The University of Hong Kong, Hong Kong; ^53^ Department of Surgery and Cancer Genetics Center, Hong Kong Sanatorium and Hospital, Hong Kong; ^54^ Department of Surgery, Ulsan University College of Medicine and Asan Medical Center, Seoul, South Korea; ^55^ Department of Gynecology and Obstetrics, University of Munich, Campus Großhadern, Munich, Germany; ^56^ Division of Gynaecology and Obstetrics, Klinikum rechts der Isar der Technischen Universität München, Munich, Germany; ^57^ Yorkshire Regional Genetics Service, Leeds Teaching Hospitals NHS Trust, Leeds, United Kingdom; ^58^ Cancer Genetics Service, National Cancer Center, Singapore, Singapore; ^59^ Lee Kong Chian School of Medicine, Nanyang Technological University, Singapore, Singapore; ^60^ Department of Preventive Medicine, Seoul National University College of Medicine, Seoul, South Korea; ^61^ Integrated Major in Innovative Medical Science, Seoul National University College of Medicine, Seoul, South Korea; ^62^ Cancer Research Institute, Seoul National University, Seoul, South Korea; ^63^ Molecular Diagnostics, Aalborg University Hospital, Aalborg, Denmark; ^64^ Clinical Cancer Research Center, Aalborg University Hospital, Aalborg, Denmark; ^65^ Department of Clinical Medicine, Aalborg University, Aalborg, Denmark; ^66^ Faculty of Medicine, School of Women's and Children's Health, University of NSW Sydney, Sydney, New South Wales, Australia; ^67^ Adult Cancer Program, Lowy Cancer Research Center, University of NSW Sydney, Sydney, New South Wales, Australia; ^68^ Clinical Genetics, Karolinska Institutet, Stockholm, Sweden; ^69^ Department of Basic Sciences, Shaukat Khanum Memorial Cancer Hospital and Research Center (SKMCH & RC), Lahore, Pakistan; ^70^ Department of OB/GYN and Comprehensive Cancer Center, Medical University of Vienna, Vienna, Austria; ^71^ Institute of Medical and Human Genetics, Charité–Universitätsmedizin Berlin, Berlin, Germany; ^72^ Faculty of Medicine Carl Gustav Carus, Institute for Clinical Genetics, TU Dresden, Dresden, Germany; ^73^ Centro de Investigación en Red de Enfermedades Raras (CIBERER), Madrid, Spain; ^74^ Fundación Pública Galega Medicina Xenómica, Santiago De Compostela, Spain; ^75^ Instituto de Investigación Sanitaria de Santiago de Compostela, Santiago De Compostela, Spain; ^76^ Institute of Human Genetics, Hannover Medical School, Hannover, Germany; ^77^ Faculty of Medicine, Center for Molecular Medicine Cologne (CMMC), University Hospital Cologne, University of Cologne, Cologne, Germany; ^78^ Clinical Cancer Genetics Program, Division of Human Genetics, Department of Internal Medicine, The Comprehensive Cancer Center, The Ohio State University, Columbus, OH; ^79^ West Midlands Regional Genetics Service, Birmingham Women's Hospital Healthcare NHS Trust, Birmingham, United Kingdom; ^80^ Department of Clinical Pathology, The University of Melbourne, Parkville, Victoria, Australia; ^81^ Cancer Epidemiology Division, Cancer Council Victoria, Melbourne, Victoria, Australia; ^82^ Department of Tumour Biology, INSERM U830, Paris, France; ^83^ Université Paris Descartes, Paris, France; ^84^ Institute of Human Genetics, University Hospital Heidelberg, Heidelberg, Germany; ^85^ Department of Surgery, Faculty of Medicine, University of Malaya, Kuala Lumpur, Malaysia; ^86^ Department of Epidemiology, Mailman School of Public Health, Columbia University, New York, NY; ^87^ Program in Cancer Genetics, Departments of Human Genetics and Oncology, McGill University, Montréal, QC, Canada; ^88^ Department of Medical Genetics, University of Cambridge, Cambridge, United Kingdom; ^89^ Department of Cancer Biology and Genetics, The Ohio State University, Columbus, OH; ^90^ Institute of Human Genetics, Pontificia Universidad Javeriana, Bogota, Colombia; ^91^ Department of Medicine, Hematology/Oncology, Goethe-University Frankfurt, Frankfurt, Germany; ^92^ Department of Gynaecology and Obstetrics, University Hospital Ulm, Ulm, Germany; ^93^ Institute of Human Genetics, University Regensburg, Regensburg, Germany; ^94^ Institute of Clinical Human Genetics, University Hospital Regensburg, Regensburg, Germany

## Abstract

To provide precise age-specific risk estimates of cancers other than female breast and ovarian cancers associated with pathogenic variants (PVs) in *BRCA1* and *BRCA2* for effective cancer risk management.

**METHODS:**

We used data from 3,184 *BRCA1* and 2,157 *BRCA2* families in the Consortium of Investigators of Modifiers of *BRCA1/2* to estimate age-specific relative (RR) and absolute risks for 22 first primary cancer types adjusting for family ascertainment.

**RESULTS:**

*BRCA1* PVs were associated with risks of male breast (RR = 4.30; 95% CI, 1.09 to 16.96), pancreatic (RR = 2.36; 95% CI, 1.51 to 3.68), and stomach (RR = 2.17; 95% CI, 1.25 to 3.77) cancers. Associations with colorectal and gallbladder cancers were also suggested. *BRCA2* PVs were associated with risks of male breast (RR = 44.0; 95% CI, 21.3 to 90.9), stomach (RR = 3.69; 95% CI, 2.40 to 5.67), pancreatic (RR = 3.34; 95% CI, 2.21 to 5.06), and prostate (RR = 2.22; 95% CI, 1.63 to 3.03) cancers. The stomach cancer RR was higher for females than males (6.89 *v* 2.76; *P* = .04). The absolute risks to age 80 years ranged from 0.4% for male breast cancer to approximately 2.5% for pancreatic cancer for *BRCA1* carriers and from approximately 2.5% for pancreatic cancer to 27% for prostate cancer for *BRCA2* carriers.

**CONCLUSION:**

In addition to female breast and ovarian cancers, *BRCA1* and *BRCA2* PVs are associated with increased risks of male breast, pancreatic, stomach, and prostate (only *BRCA2* PVs) cancers, but not with the risks of other previously suggested cancers. The estimated age-specific risks will refine cancer risk management in men and women with *BRCA1/2* PVs.

## INTRODUCTION

It is well established that pathogenic variants (PVs) in *BRCA1* and *BRCA2* (*BRCA1/2*) are associated with increased risks of breast and ovarian cancers in women for which reliable risk estimates are available.^1^ Accumulated evidence indicates that *BRCA1/2* PVs are also associated with pancreatic cancer^2-8^ and male breast cancer risks^3,6,9-13^ and that *BRCA2* PVs are associated with prostate cancer risk, particularly aggressive prostate cancer, whereas the association between *BRCA1* PVs and prostate cancer risk is still debated.^2,5,6,8,14-17^ Associations with risks for other cancers have also been suggested, including colorectal, liver, and stomach cancers for *BRCA1/2* PVs; cervical, corpus uteri, kidney, and testis cancers for *BRCA1* PVs;^3,4,6,8,18,19^ and bone, brain, blood, and gallbladder cancers and malignant melanoma for *BRCA2* PVs.^2,5,6,8,20^ However, these associations are based on studies with relatively small sample sizes, resulting in imprecise cancer risk estimates.

**CONTEXT**

**Key Objective**
The associations of pathogenic variants (PVs) in *BRCA1* and *BRCA2* with cancers other than female breast and ovarian cancers remain uncertain. Precise risk estimates are required to inform effective cancer risk management. This study investigates the associations between the risks of 22 cancers and *BRCA1/2* PVs using data from 5,341 families segregating *BRCA1* or *BRCA2* PVs.
**Knowledge Generated**
*BRCA1* and *BRCA2* PVs are associated with increased risks of male breast, pancreatic, and stomach cancers; male *BRCA2* carriers are also at increased prostate cancer risk. No associations were found with risks of other cancers. The cumulative risks to age 80 years ranged from 0.4% for male breast cancer to approximately 2.5% for pancreatic cancer for *BRCA1* carriers and from approximately 2.5% for pancreatic cancer to 27% for prostate cancer for *BRCA2* carriers.
**Relevance**
The findings provide age-specific cancer risk estimates and will allow for improved cancer risk assessment of male and female carriers.

The National Comprehensive Cancer Network and other guidelines recommend breast and ovarian cancer screening for *BRCA1*/*2* carriers and prostate cancer screening particularly for *BRCA2* carriers. Notably, National Comprehensive Cancer Network guidelines recently addressed testing and management for pancreatic cancer risk in *BRCA1/2* carriers, but only in the presence of a positive family history of the disease.^21,22^ Overall, current guidelines suggest that men and women with *BRCA1/2* PVs should consider participation in investigational screening studies and receive education regarding signs and symptoms of cancers possibly associated with *BRCA1/2* PVs.^21^ The availability of more precise risk estimates will aid translation into evidence-based clinical guidelines for the cancer risk management in *BRCA1/2* carriers and may guide treatment options for patients with cancer.

To inform clinical management strategies and optimize guidelines for cancer risk management in female and male *BRCA1/2* carriers, we comprehensively assess the associations of *BRCA1/2* PVs with risks of 22 cancers, other than female breast and ovarian cancers.

## METHODS

### Study Sample

Data on 7,618 families with at least one family member having a *BRCA1* or *BRCA2* PV were obtained from 26 study groups in the Consortium of Investigators of Modifiers of *BRCA1*/2 (Data Supplement, online only).^23^ Only families with a clearly PV identified were included.^24^ The majority of families (7,281) were ascertained through an index individual attending cancer family clinics, mainly because of having multiple affected relatives, and 337 families were ascertained through an index case with breast or ovarian cancer, unselected for family history. All index individuals were age ≥ 18 years. For each family member, data including familial relationship, *BRCA1/2* PV status, sex, year of birth, and years or age at pedigree data collection, death, and cancer diagnoses were collected (Data Supplement). All participants provided written informed consent and participated in studies at the host institutions under ethically approved protocols.

### Statistical Analysis

*BRCA1* and *BRCA2* families were analyzed separately. Complex segregation analysis,^25^ which considered the observed phenotype and observed or inferred genotype information of all family members, was used to estimate relative risks (RRs) for 22 first primary cancer sites, excluding female breast and ovarian cancers (Table 1). This involved comparing the observed cancer incidences for carriers with the age-, country- and birth cohort-specific population incidences (Cancer Incidence in Five Continents^26^); thus, the estimated RRs were equivalent to standardized incidence ratios. Noncarriers were assumed to develop the cancers according to population incidences. Pedigree likelihoods were constructed and maximized using the pedigree analysis software MENDEL.^27^

**TABLE 1.**
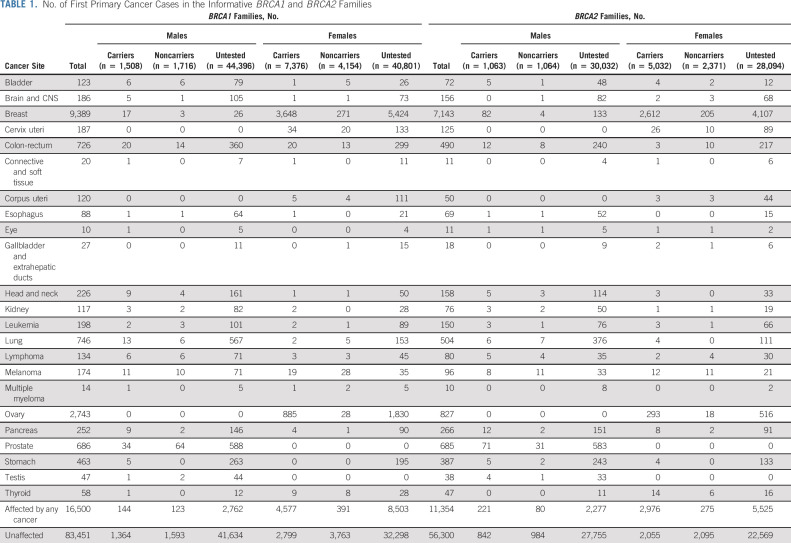
No. of First Primary Cancer Cases in the Informative *BRCA1* and *BRCA2* Families

Individuals were followed from birth until the age of the first primary cancer diagnosis, death, age at pedigree-data collection, risk-reducing mastectomy and/or salpingo-oophorectomy (if these occurred at least 1 year before breast or ovarian cancer diagnoses, respectively), or age 80 years, whichever occurred first. Missing year of birth and cancer diagnosis age were imputed (Data Supplement).

Each individual was assumed to be at risk of developing the cancer of interest, as well as breast or ovarian cancer. The RRs for female breast and ovarian cancers were assumed to be equal to previous estimates^28^; therefore, we only estimated the RR for the cancer of interest. We fitted models in which the RRs were assumed to be constant with age, birth cohort, sex, and study group and separate models with sex-specific RRs. For cancers with significant associations, we investigated whether the RRs varied by age. RRs from the best fitting models were used to estimate age-specific absolute risks on the basis of UK cancer incidences in year 2008-2012 (Data Supplement).

Because family ascertainment varied across study groups, we adjusted for the ascertainment of each family separately using an ascertainment-assumption-free approach.^29-31^ Pedigree likelihoods were computed conditional on any data that may be relevant to the ascertainment (Data Supplement). Noninformative families, in which no additional information beyond the data relevant to the ascertainment was available, were excluded from analysis. Since cancer family history was self-reported, we assessed the possibility of systematic under-reporting of specific cancers at the individual study group level and excluded any study groups in which under-reporting was likely relative to the population incidences (Data Supplement).

Sensitivity analyses under alternative inclusion, censoring, or ascertainment assumptions were performed for cancers that demonstrated associations: (1) stratifying by geographical region (Asian countries *v* others); (2) including study groups with possible cancer under-reporting; (3) excluding individuals with missing age at diagnosis; (4) individuals with risk-reducing bilateral mastectomy and/or salpingo-oophorectomy were still considered to be at risk of developing the other cancers, except breast and ovarian cancers; and (5) assuming the data relevant to the ascertainment for clinic-based families do not include the family history of cancer of interest. To account for population differences in melanoma skin pigmentation, we also conducted sensitivity analyses for melanoma by using (1) only families from Australia, Northern Europe, and North America; (2) only families in which probands self-identified as White European; and (3) only the families satisfying both (1) and (2).

All statistical tests were two-sided, and associations with a nominal *P* < .05 were considered statistically significant.

## RESULTS

After ascertainment adjustment, 3,184 *BRCA1* families and 2,157 *BRCA2* families were informative for inclusion in the analysis, including 14,979 carriers, 9,296 noncarriers, and 153,323 untested individuals (Data Supplement). 61.3% of probands had self-reported ethnicity data. Of those, 77.0%, 11.5%, 4.7%, 3.3%, and 1.2% self-identified as White European, Asian, Ashkenazi Jewish, Hispanic, and Black, respectively. Prostate, lung, colorectal, stomach, and pancreatic cancers were the most common cancers in the data set, aside from breast and ovarian (Table 1). The age at diagnosis for each cancer by PV status is shown in the Data Supplement. After excluding study groups in which there was potential cancer under-reporting (Data Supplement), the proportions of families included in the estimation of cancer-specific risks varied from approximately 15% for lymphoma and multiple myeloma to > 90% for pancreatic and male breast cancers (Data Supplement).

### Cancer Associations With *BRCA1* PVs

*BRCA1* PVs were associated with male breast (RR = 4.30; 95% CI, 1.09 to 16.96), gallbladder (RR = 3.34; 95% CI, 1.34 to 8.28), pancreatic (RR = 2.36; 95% CI, 1.51 to 3.68), stomach (RR = 2.17; 95% CI, 1.25 to 3.77), and colorectal (RR = 1.48; 95% CI, 1.01 to 2.16) cancers (Table 2). No association was found for prostate cancer (RR = 0.82; 95% CI, 0.54 to 1.27). No difference in the RR estimates by sex was observed for any of the 17 non–sex-specific cancers (all *P* > .07; Table 3).

**TABLE 2.**
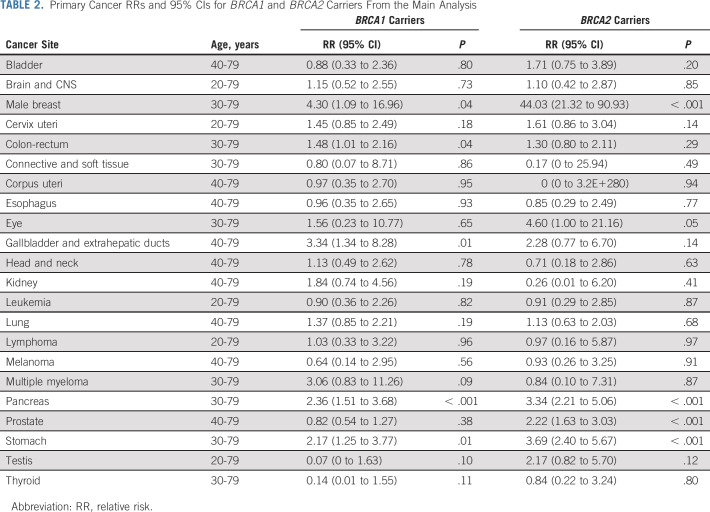
Primary Cancer RRs and 95% CIs for *BRCA1* and *BRCA2* Carriers From the Main Analysis

**TABLE 3.**
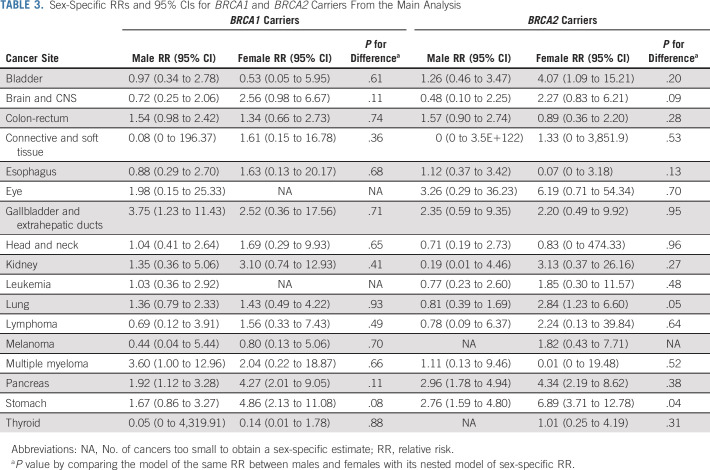
Sex-Specific RRs and 95% CIs for *BRCA1* and *BRCA2* Carriers From the Main Analysis

A model with RRs stratified by age 65 years (Data Supplement) provided a significantly better fit for stomach cancer: RR = 3.50 (95% CI, 2.01 to 6.10) for age < 65 years and higher than 0.61 (95% CI, 0.16 to 2.30) for age ≥ 65 years (*P*-heterogeneity = .01). For male breast cancer, a model with RRs stratified by age decade provided a better fit than the model with an age-constant RR (*P* = .03), but this was mainly driven by the lack of cases in the age group of 50-59 years (Data Supplement).

### Cancer Associations With *BRCA2* PVs

*BRCA2* PVs were associated with increased risks of male breast (RR = 44.0; 95% CI, 21.3 to 90.9), stomach (RR = 3.69; 95% CI, 2.40 to 5.67), pancreatic (RR = 3.34; 95% CI, 2.21 to 5.06), and prostate (RR = 2.22; 95% CI, 1.63 to 3.03) cancers (Table 2). Female carriers had a higher risk of stomach cancer (RR = 6.89; 95% CI, 3.71 to 12.78) than male carriers (RR = 2.76; 95% CI, 1.59 to 4.80; *P*-heterogeneity = .04; Table 3).

A model with RRs stratified by age 65 years (Data Supplement) provided a significantly better fit for pancreatic cancer: RR = 4.92 (95% CI, 2.96 to 7.80) for age < 65 years and higher than 1.77 (95% CI, 0.87 to 3.58) for age ≥ 65 years (*P*-heterogeneity = .03). There was a suggestion that the prostate cancer RR was greater for age < 65 years (RR = 3.10; 95% CI, 2.00 to 4.79) than age ≥ 65 years (RR = 1.69; 95% CI, 1.09 to 2.62), but this model did not fit significantly better than the model with an age-constant RR (*P* = .06).

### Sensitivity Analysis

The results are described in detail in the Data Supplement. There was no significant difference in the RR estimates by geographical region. The observed cancer associations were robust to all sensitivity analyses, except for colorectal and gallbladder cancers. No association was found for melanoma even when analyses were restricted to families from Australia, Northern Europe, and North America or families in which probands self-identified as White European.

### Absolute Risks

**FIG 1.**
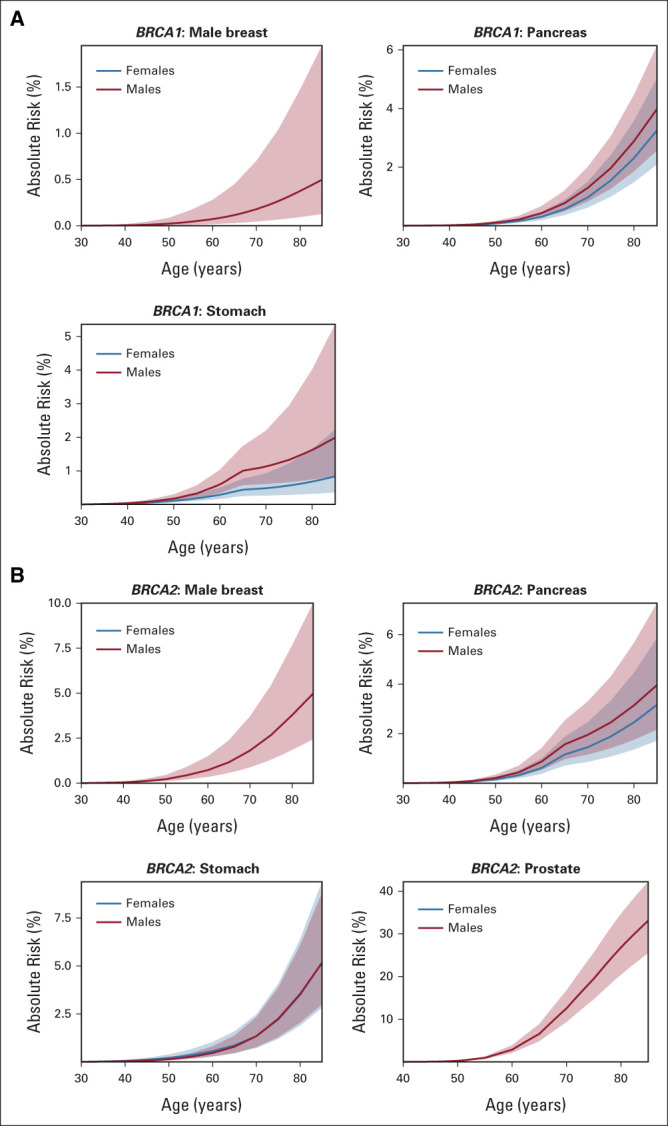
Age-specific absolute risks (%) and 95% CIs of primary cancers on the basis of UK cancer incidences in years 2008-2012 for (A) *BRCA1* and (B) *BRCA2* carriers. Solid lines are the age-specific absolute risk estimates, and ribbons are the relevant 95% CIs.

RRs from the main analysis best-fitting models were used to calculate age-specific absolute cancer risks (Table 4 and Fig 1). By age 80 years, the male breast cancer risk for *BRCA1* and *BRCA2* carriers was 0.4% (95% CI, 0.1 to 1.5) and 3.8% (95% CI, 1.9 to 7.7), respectively; the pancreatic cancer risk varied between 2.3% and 3.0% for both male and female *BRCA1* and *BRCA2* carriers; the stomach cancer risks were 1.6% (95% CI, 0.7 to 4.0) for male and 0.7% (95% CI, 0.3 to 1.7) for female *BRCA1* carriers and approximately 3.5% for both male and female *BRCA2* carriers. The prostate cancer risk associated with *BRCA2* PVs was 26.9% (95% CI, 20.5 to 34.7) by age 80 years and 33.1% (95% CI, 25.5 to 42.2) by age 85 years.

**TABLE 4.**
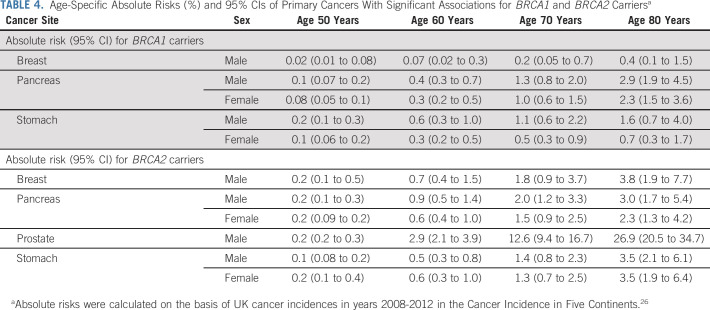
Age-Specific Absolute Risks (%) and 95% CIs of Primary Cancers With Significant Associations for *BRCA1* and *BRCA2* Carriers^a^

## DISCUSSION

This study assessed the risks associated with *BRCA1*/2 PVs for 22 first primary cancers, other than female breast and ovarian cancers, and further clarified the cancer spectrum associated with *BRCA1*/*2* PVs.

The associations of *BRCA1/2* PVs with the risks of male breast and pancreatic cancers were confirmed and refined, as well as the association of prostate cancer with *BRCA2* PVs, regardless of age and aggressiveness.

The lifetime male breast cancer risks were previously reported to be 2%-6% for *BRCA1* and 7%-13% for *BRCA2* carriers (Data Supplement).^3,6,9-13^ We estimated these risks to be somewhat lower, 0.4% (95% CI, 0.1 to 1.5) and 3.8% (95% CI, 1.9 to 7.7), respectively. The pancreatic cancer associations were consistent with previously reported RRs of 2-3 and lifetime risks of 1%-4% for *BRCA1* carriers^3,4,6^ and RRs of 3-6 and lifetime risks of 3%-5% for *BRCA2* carriers (Data Supplement).^2,5-8^ Notably, the RR was higher for *BRCA2* carriers age < 65 years.

Previous retrospective studies reported prostate cancer RRs of 2-6 and absolute risks of 17%-31% by age 80 years for *BRCA2* carriers (Data Supplement).^2,5,6,8,14-17^ Our estimated absolute risk by age 85 years was 33%, lower than the recently reported prospective estimate of 60% by Nyberg et al.^32^ However, after adjusting for possible increased prostate-specific antigen screening effects in the prospective study, their estimate was 41% (95% CI, 22 to 59), consistent with our estimate. The present estimate is unlikely to be subject to increased screening biases since prostate cancer family history was retrospectively collected, and increased screening in relatives is unlikely to have taken place before the identification of *BRCA2* PVs. The reported associations of *BRCA1* PVs with prostate cancer risk are inconsistent, with RRs of 0.4-4, most not statistically significant.^3,4,6,8,14-18,32,33^ This study confirms that *BRCA1* PVs are not associated with overall prostate cancer risk.

Among the suggested associations with other cancers, the association between *BRCA1/2* PVs and stomach cancer is under considerable debate.^4,5^ This study validated and further elucidated this association: there were associations with both *BRCA1* and *BRCA2* PVs, with RRs of 2.17 (3.50 for age < 65 years) and 3.69, respectively. Our estimates better refined the previously reported RRs of 2-7 for *BRCA1* carriers^3,6,8^ and approximately 2.6 for *BRCA2* carriers (Data Supplement).^2,8^ Notably, our findings showed that the stomach cancer RR for female *BRCA2* carriers was higher than the estimate for male carriers although this translated in similar absolute risks, given the higher incidence of male stomach cancer in the general population. However, we cannot exclude the possibility that the higher female RR may be due to the misclassification of some ovarian cancers as stomach cancers.^2,34^

Data in the current study come from either epidemiologic studies or families undergoing PV screening collected at genetics centers. Although individual studies and clinical genetic centers, where possible, confirmed reported cancer diagnoses in families through medical records or registries as part of standard clinical practice, cancer confirmation information is not available centrally and it was not feasible to collect this at such a large scale. However, a key advantage of the present study is the large sample size, which results in RR estimates with greater precision. Only a small number of family-based studies reported cancer confirmation rates.^2,4,5,8^ Our RR estimates for stomach cancer, which may be susceptible to a greater degree of misclassification bias than other cancers,^2,34^ are not significantly different from the estimates from studies that reported cancer confirmation. However, the present RRs have similar or greater precision than the published estimates from studies with high cancer confirmation rates (Data Supplement).

In the present study, previously suggested associations of *BRCA1/2* PVs with risks of other genitourinary cancers and melanoma^2,8^ were not replicated. Although associations of *BRCA1* PVs with colorectal and gallbladder cancers were observed, the results were not robust in the sensitivity analyses performed.

Increased risks of bone and liver cancer have also been reported for *BRCA1* or *BRCA2* carriers.^4-6^ However, liver and bone are common metastatic sites for breast, prostate, or pancreatic cancers and could be the presenting cancer. Since no pathology confirmation data were available, we did not examine these associations in the main analysis. If we assume that the reported bone and liver cancers in the data set are indeed first primaries, the data suggest no association with *BRCA1* PVs, but that *BRCA2* carriers are at seven-fold increased risk of bone cancer and five-fold increased risk of liver cancer without significant differences between males and females (Data Supplement). However, no conclusion for these associations can be drawn without pathology confirmation.

Overall, the estimated age-specific relative and absolute risks suggest that, in addition to breast and ovarian cancers, the clinical management of *BRCA1/2* carriers should focus on cancer sites, which now show robust associations, such as prostate (*BRCA2* carriers only), pancreatic, and possibly stomach cancers. Notably, although rare, pancreatic and stomach cancers are associated with poor prognosis and their incidences have been rising over time, and thus, our results highlight the importance of screening for upper gastrointestinal tract malignancies for *BRCA1* and *BRCA2* carriers, particularly for age < 65 years. On the other hand, some cancers previously taken into consideration for screening for *BRCA1/2* carriers, like melanoma, may be reconsidered, to further optimize cancer prevention screening strategies and eventually reduce carriers' distress. Given that the cancer risk associations were found for both male and female carriers, the results also suggest that male relatives of known *BRCA1/2* carriers should be informed about their individual cancer risk and encouraged to be tested.^35,36^ It has been shown that knowing the germline *BRCA1/2* PV status can influence treatment options for patients with cancer, leading to improved prognosis. For example, poly (ADP-ribose) polymerase inhibitor therapies that have been used successfully in the treatment of *BRCA*-related breast and ovarian cancers^37^ are now beginning to be used for pancreatic and prostate cancers,^38,39^ and in the near future, they might also be used for stomach cancer.^40^

To avoid biases in the risk estimates related to the ascertainment of clinic-based families, on the basis of multiple affected family members, we used a conservative ascertainment adjustment approach by conditioning on the family histories of cancers of breast and ovary and the cancer site under investigation. When only family history of female breast and ovarian cancers was considered in the ascertainment, the RR estimates were somewhat higher for most cancers but with narrower CIs (Data Supplement). Therefore, conditioning on the family history of the cancer of interest is unlikely to have led to substantial underestimation of risk. A notable exception was male breast cancer, where much higher RR estimates were obtained. However, this estimate is most likely biased because male breast cancer family history has been an important factor in considering *BRCA1/2* germline genetic testing since the discovery of *BRCA1/2*.

This study has several limitations. First, this is a retrospective family-based study, with self-reported cancer family history, which may be inaccurate.^41,42^ Second, 7%-40% of reported cancer cases had missing age at diagnosis, with stomach cancer having the largest proportion. To minimize these potential biases, we performed sensitivity analyses excluding any study groups in which under-reporting was likely and any cases with missing age at diagnosis, and conclusions remained similar for most cancers. Third, we presented our results without any multiple testing adjustment. However, even using a false discovery rate adjustment, all the observed associations for *BRCA2* carriers and the pancreatic cancer association for *BRCA1* carriers had false discovery rates < 0.05. Fourth, the ethnicity of the family proband was not systematically collected by all studies because of variations in local data collection protocols. Among those with recorded ethnicity, in Asia-based studies, 97.7% of probands were Asian and in the rest of the studies 86.1%, 5.2%, 3.7%, 1.3%, and 1.1% of probands were White European, Ashkenazi, Hispanic, Black, and Asian, respectively. Therefore, the power to investigate the associations by all ethnic groups was limited. However, we did not find evidence of heterogeneity in the RRs by geographical region (Asia *v* others). Whether our risk estimates are applicable to non-European populations requires further investigation. Fifth, we did not have data on other genetic and environmental factors, so we were unable to investigate the modification effects of these factors; therefore, our risk estimates should be interpreted as the average risks across all potential genetic and environmental modifiers.

In conclusion, this study confirms that, aside from female breast and ovarian cancers, *BRCA1/2* PVs are associated with increased risks of breast cancer in men, and pancreatic and stomach cancers in both sexes, and that only *BRCA2* carriers are at elevated prostate cancer risk. *BRCA1/2* PVs were not associated with the risks of any other cancers previously suggested. The association results and estimated age-specific risks will improve the cancer risk management for men and women with *BRCA1/2* PVs.
